# BREAST-Q Measurement of the Patient Perspective in Oncoplastic Breast Surgery: A Systematic Review

**DOI:** 10.1097/GOX.0000000000001904

**Published:** 2018-08-07

**Authors:** Liang Q. Liu, Olivier A. Branford, Sinead Mehigan

**Affiliations:** From the * Department of Adult, Child & Midwifery, School of Health and Education, Centre for Critical Research in Nursing and Midwifery, Middlesex University, The Burroughs, London, United Kingdom; † Department of Plastic Surgery, Queen Victoria Hospital, West Sussex, United Kingdom.

## Abstract

**Background:**

Since BREAST-Q was developed in 2009, it has been widely used by clinicians and researchers to capture information regarding health-related quality of life (HRQoL) and patient satisfaction related to breast surgery. Yet clinical guidelines regarding the use of BREAST-Q for assessment of success of surgery in women with breast cancer remain limited. To maximize the benefits of using BREAST-Q to inform clinical decision making, this systematic review aimed to identify and appraise current evidence on patient-reported outcomes (PROs) assessed by BREAST-Q associated with breast oncoplastic surgery.

**Methods:**

A detailed search strategy was implemented and electronic databases searched include PubMed, MEDLINE, CINAHL, and PsycINFO. Review was limited to peer-reviewed studies published in English from 2009 to January 2018. Any interventional and observational studies that used BREAST-Q to assess PROs in the assessment of breast oncoplastic surgery were included.

**Results:**

Fifty-four peer-reviewed articles met inclusion criteria. Fifty-three studies were observational, 1 study was interventional. Current comparative studies using BREAST-Q indicated that abdominal flap, buttock flap, or thigh flap reconstruction offered highest satisfaction with breast; contralateral prophylactic mastectomy with immediate reconstruction offered higher levels of satisfaction with breast, but poor postsurgical physical well-being. Silicone implant and no radiation therapy offered higher level satisfaction and HRQoL.

**Conclusions:**

Current evidence showed that BREAST-Q can effectively measure patient’s satisfaction and HRQoL in relation to different type of breast oncoplastic surgeries. BREAST-Q captured meaningful and reliable information from the patients’ perspective and may be useful for clinical decision making.

## Abstract 2

Supplemental Digital Content is available in the text.

## INTRODUCTION

Over 50,000 women are diagnosed with breast cancer annually in the United Kingdom, 30–40% require mastectomy.^1,2^ Advances in breast cancer diagnosis and management have produced significant improvements in breast cancer–related survival. Over 60% of women who had breast cancer survive for 20 years following initial diagnosis.^3,4^ Therefore, there is an increased need to recognize quality of life and quality of care in this population. Clinicians need to consider patient perceptions of the delivery of care and results of surgery in addition to patient morbidity and mortality. Measuring patient-reported outcomes (PROs) has become increasingly important in cancer care.^5^ Patients’ satisfaction with their surgical outcome alongside postsurgery quality of life and psychosocial wellbeing is vitally important for women undergoing breast oncoplastic surgery. Indeed PROs are increasingly important to the modern model of breast cancer management with potential for greatly enhanced quality of care, reflecting the change of focus from the physician’s to the patient’s perspective. It is suggested that the use of valid, reliable, and clinically useful patient-reported outcome measures (PROM’s) should have an increased role in decision making.^6,7^

For instance, Pusic et al.^8^ conducted a systematic review of PRO instruments for measuring health-related quality of life (HRQoL) following cosmetic and reconstructive breast surgery. They found that only 1 breast-related symptoms questionnaire demonstrated adequate development and validation in its target population. Furthermore, that questionnaire focusing on measuring breast symptoms only had significant content limitations.^8^ Pusic et al.^8^ concluded that a new validated cosmetic and reconstructive breast surgery–specific instrument was needed to determine the benefits of breast surgery. Subsequently, Pusic et al.^9^ developed a conceptual framework, BREAST-Q, drawing on patient interviews, focus groups, expert panels, and a literature review. BREAST-Q measures patients’ perceptions both quantitatively and qualitatively by examining 3 quality of life domains (psychosocial well-being, physical well-being, and sexual well-being) and 3 satisfaction domains (satisfaction with breasts, satisfaction with outcome, and satisfaction with care).^9^ BREAST-Q is free of charge for nonprofit academic research and in clinical care. Since its inception, BREAST-Q has been translated into 30 languages and widely used by clinicians to gain insights into the effectiveness of breast cosmetic and therapeutic surgeries. Recently, Cohen et al.^10^ carried out a scoping review on the use of BREAST-Q in surgical research, analyzing peer-reviewed articles published between 2009 and 2015 including breast cosmetic (augmentation or reduction) and therapeutic surgeries. They aimed to provide plastic surgeons with general overviews on the use of BREAST-Q from a clinical perspective. The authors made some generalized observations of how BREAST-Q has increased the use of PROMs in breast surgery. Yet, there was no specific research question addressed by that review. They found the level of satisfaction and quality of life or stress triggers in women undergoing breast oncological surgeries can be different from women who seek cosmetic breast surgeries. Although the use of PROMs has increased, it is unclear to what extent BREAST-Q has been used to assess satisfaction and quality of life associated with breast oncoplastic surgery. Clinical practice guidelines regarding the use of BREAST-Q for assessment of the success of surgery in women with breast cancer remain limited. To maximize the benefits of using BREAST-Q to inform clinical decision making, overall aim of this review was to critically appraise and synthesize the research evidence available on PROMs assessed by BREAST-Q associated with breast surgery in women who had breast cancer. This review therefore sought to address 4 specific questions:

1) To what extent has BREAST-Q been used to evaluate satisfaction and HRQoL among patients undergoing surgery for breast cancer?
2) Which types of oncoplastic surgical procedures have been studied for PROs using BREAST-Q questionnaire in women who had breast cancer?
3) What are the outcome parameters of BREAST-Q used in the literature?
4) How clinically effective is BREAST-Q as a tool for measuring PROMs in oncoplastic breast surgery?

## METHODS

### Identification of Studies

A systematic review protocol for the identification, retrieval, and appraisal of the evidence was first developed in June 2016 and updated in June 2017.^11^ The final search was carried out on January 15, 2018. We searched all relevant literature published from 2009 to January 15, 2018 in 4 databases, without any language restrictions. We used free-text, key word, and Medical Subject Headings terms for each of the following databases: PubMed, MEDLINE, CINAHL, and PsycINFO. We entered subject subheadings and word truncations according to database requirements to map all possible key word terms. Search terms included

- Search terms for breast cancer: breast cancer or mammary cancer
- Search terms for breast oncology surgery: breast surgery, lumpectomy, breast conservation, breast conserving surgery, mastectomy, breast reconstruction, questionnaires
- Search terms for quality of life and patient satisfaction measurement tool: BREAST-Q. QoL, HRQoL, outcome assessment, outcomes and process assessment, patient reported outcome, health status, and satisfaction.

We also searched the NICE, Scottish Intercollegiate Guidelines Network. Additionally, we checked reference lists of included studies and other relevant review papers for further eligible studies.

### Inclusion Criteria

To capture all relevant evidence, eligible studies included experimental studies (randomized controlled trials, nonrandomized controlled trial, and pre- and poststudies) and observational studies (cohort studies, case series, case control studies). Further study inclusion criteria were applied as follows:

- Primary research studies published in peer-reviewed journals;
- Studies with a target population including women with primary breast cancer irrespective of their age, type, and stage of cancer, that is, invasive or in situ;
- Studies comparing any type of oncoplastic breast surgery, for example, breast conservative surgery, mastectomy with/without any type of reconstruction;
- Studies reporting outcomes using BREAST-Q questionnaires developed by Pusic et al (2009).^9^

We excluded literature reviews, book chapters, conference proceedings, dissertations/thesis, letters or editorial opinions, and non-English articles. We also excluded if target populations were patients who did not have breast cancer. Types of studies excluded were case reports or any study without comparison of oncological breast therapies. Studies that did not use BREAST-Q questionnaire or did not fully report BREAST-Q satisfaction or HRQoL outcomes were also excluded.

### Data Extraction and Analysis

A data extraction form of common tables was designed and piloted in line with the aim and objectives of this review. The following data were extracted from eligible articles by 1 reviewer (L. Q. L.) and verified by a second reviewer (S. M.) to ensure accuracy: year of publication, country of author affiliated, sample size, participants’ age, the type of oncological surgery, follow-up period, outcomes measured by BREAST-Q and findings.

Unlike experimental studies, there are no validated assessment tools to assess the quality of observational studies so far. As most of the articles in our review were observational, each reviewer independently reported on the following: role of the investigator (ie, interventional versus observational study), overall study design (prospective, retrospective, or cross-sectional), type of study (ie, randomized or nonrandomized clinical trial, survey, case series, etc.). Studies were graded using a hierarchy of evidence, based on the modified Oxford Centre for Evidence-Based Medicine 2011 “Levels of Evidence and Clinical Guidance Outcomes group”^12^ like Harding et al.^13^ and Clucas et al.^14^ A detailed grading scale is summarized (**see appendix, Supplemental Digital Content 1**, which displays Grading criteria for primary studies, http://links.lww.com/PRSGO/A833).

Any disparity in either selecting eligible articles or assessed findings between the 2 independent reviewers was resolved through consultation with a third reviewer (O. A. B).

## RESULTS

### Included Studies

**Fig. 1.**
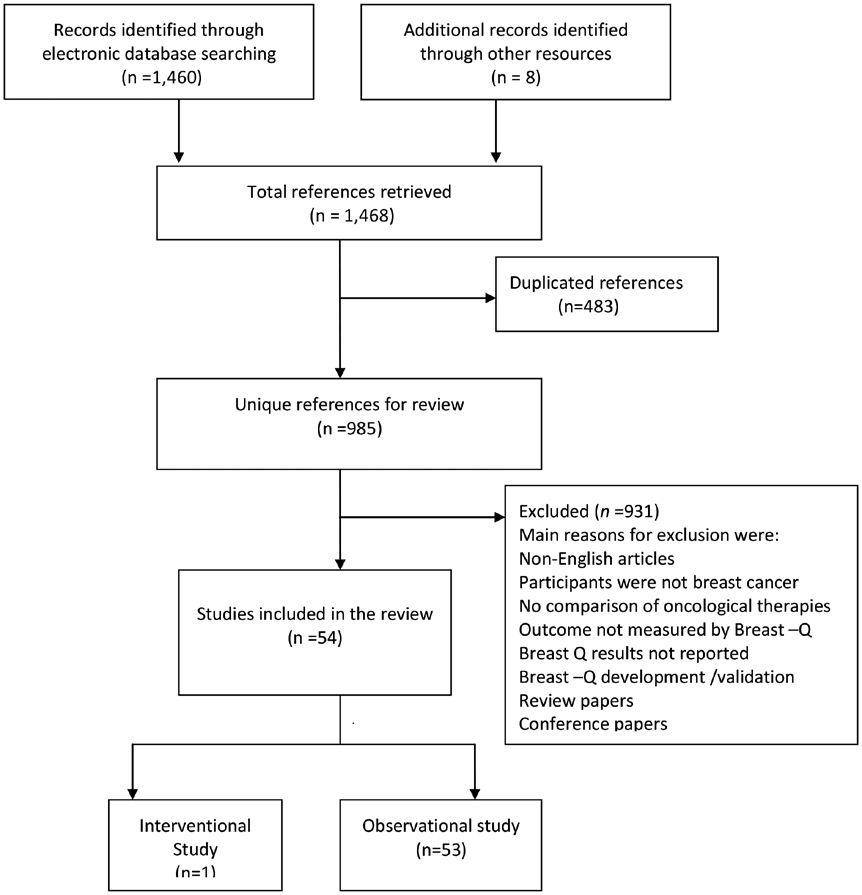
A flowchart of the selection process for articles.

**Fig. 2.**
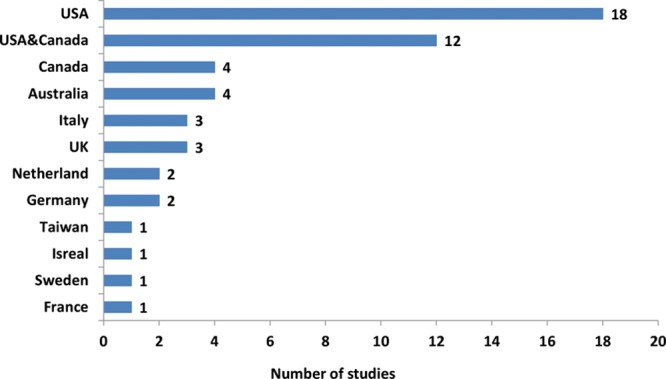
Number of studies conducted across 11 countries.

**Fig. 3.**
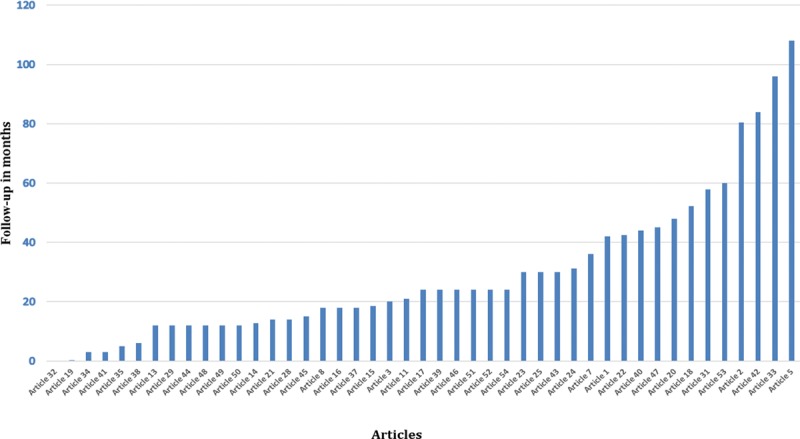
Mean duration of follow-up in 45 studies.

The literature search identified a total of 985 unique references of which 54 studies met the inclusion criteria for full data extraction (Fig. 1). All 54 articles identified their target population as patients with primary breast cancer (invasive or in situ), with sample sizes ranging from 13 to 7,619. Figure 2 shows the frequency of studies conducted across 11 countries. Nine studies did not report the follow-up duration at the time of completing BREAST-Q questionnaire. Figure 3 demonstrates the mean follow-up duration in those 45 studies reported the follow-up duration. The characteristics of all 54 studies are summarized in **Supplemental Digital Content 1**.

### Grade of Evidence

Out of 54 studies included in this review, 53 were observational studies, 1 study was experimental. Fourteen studies were classified as grade II, 12 were grade IIA (by reporting adjusted confounding factors, having a follow-up of more than 75% and key characteristics matched between comparison groups). The majority of the studies (34 of 54) were classified as grade III. The remaining 6 studies were rated as grade IV (weak evidence).

### BREAST-Q Modules

The BREAST-Q tool consists of different modules to measure patients reported outcomes following surgery. There are breast cancer–specific modules (mastectomy, reconstruction, and breast conserving-therapy), in addition to modules for noncancer surgery (breast reduction/mastopexy, breast augmentation). The modules and individual domains of the BREAST-Q questionnaire were selected by authors with diverse aims described across the 54 studies reviewed. The use of different outcome domains together with varied breast oncological therapy/procedures and different study designs limits the comparability of results across different samples. This inevitably prevented meta-analysis of the data.

**Fig. 4.**
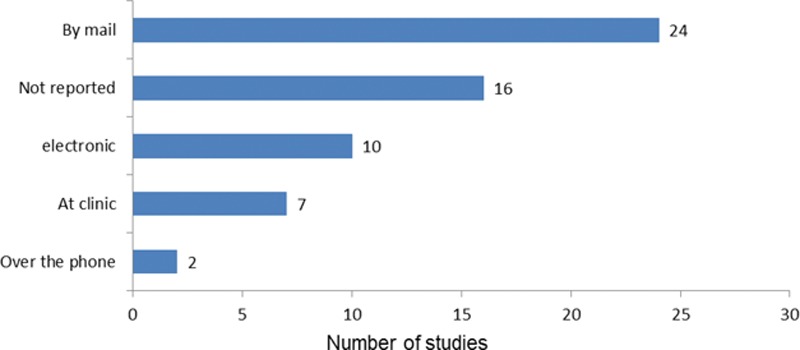
Frequency of distribution method of BREAST Q questionnaires.

All 54 studies aimed to assess satisfaction and/or quality of life after breast oncological therapy. Figure 4 demonstrates how the questionnaire was distributed. All the studies used the reconstruction module to measure patient-reported outcomes, with 3 studies comparing masterectomy alone versus mastectomy plus reconstruction. Therefore, mastectomy module was used for patients who had mastectomy alone in those 3 studies (**see appendix, Supplemental Digital Content 2**, articles 3, 10, and 16, which displays characteristics of all 54 articles included in this review, http://links.lww.com/PRSGO/A834). About 3 quarters of the studies (37 of 54) assessed postsurgery outcomes alone, 17 studies reported both pre- and postsurgery outcomes, and 2 studies reported presurgery outcomes only.

Although all studies measured satisfaction and/or quality of life, 5 reported satisfaction outcomes only (**Supplemental Digital Content 2** articles 2, 6, 13, 25, and 40), and 3 reported on the quality of life domain alone (**Supplemental Digital Content 2** articles 20, 26, and 27) ^35,41,42^ All other 46 studies reported both satisfaction and HRQoL domains. Satisfaction with breast was measured in all 54 studies. Other satisfaction domains such as satisfaction with care, that is, surgery outcome, medical staff, surgeon, information, and office staff were selectively reported by individual studies.

### BREAST-Q Response Rate

**Fig. 5.**
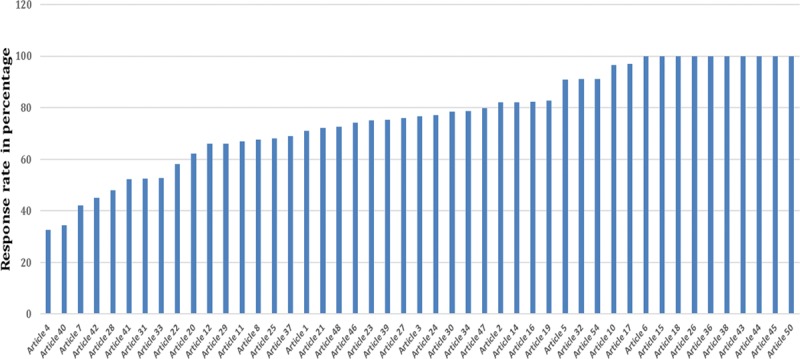
Response rate of BREAST-Q questionnaire.

Thirty-eight studies reported a response rate for completion of the BREAST-Q, which ranged from 32% to 100% (Fig. 5). One study noted a lower response rate in response to the sexual well-being domain in comparison with other domains (**Supplemental Digital Content 2** article 13).

### Comparison of Models for Breast Cancer Treatment

A broad range of oncoplastic surgical procedures were described by individual studies. All 54 studies described comparisons of different breast cancer treatments/surgical procedures. Comparisons included conservative surgery versus mastectomy with reconstruction versus mastectomy only; mastectomy with immediate reconstruction versus delayed reconstruction; symmetrization procedures versus no/delayed symmetrization; nipple-sparing mastectomy versus skin-sparing mastectomy; autologous grafting versus implant reconstruction; silicone versus saline implant, shaped gel versus round gel implant, radiation therapy versus no radiation therapy, comparison among different type of reconstruction procedures, that is, tissue expander/implant, direct to implant, microsurgical flaps (transverse rectus abdominis myocutaneous, muscle-sparing transverse rectus abdominis myocutaneous, deep inferior epigastric artery perforator, superficial inferior epigastric artery, superior gluteal artery perforator, and inferior gluteal artery perforator, and pedicled transverse rectus abdominis myocutaneous flaps (and lat dorsi).

### Patient-reported Outcomes as Measured by BREAST-Q

#### Satisfaction and HRQoL Domains

Table 1 draw together a summary of comparative assessments of satisfaction with the breast and HRQoL after different breast oncological therapies, as reported in those studies within this review. Given that the comparison mode varied greatly across individual studies, the data in Table 1 may give indications, rather than definitive guides for clinical decisions.

**Table 1.**
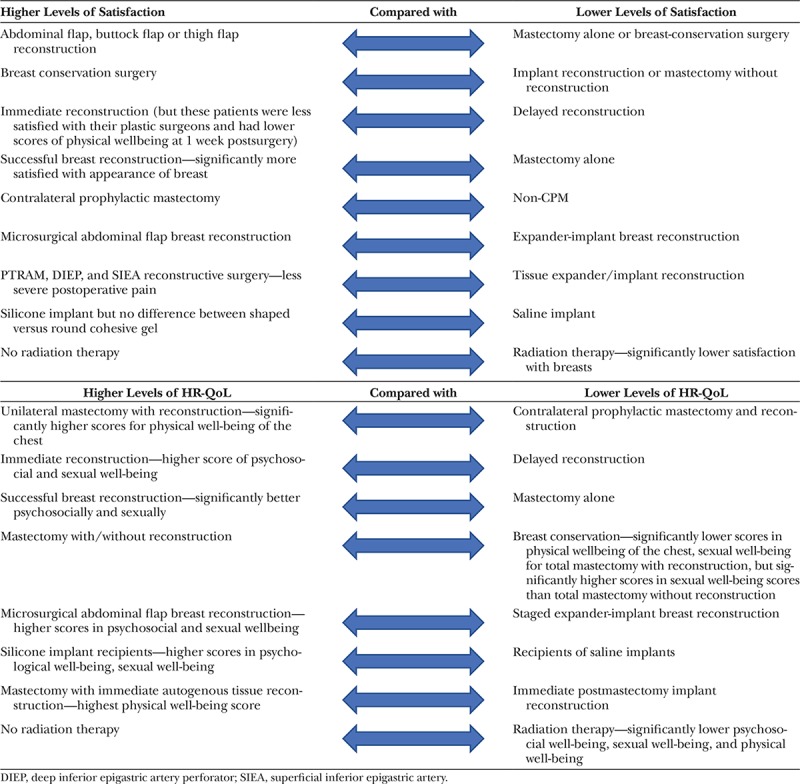
Summary of Satisfaction and HR-QoL Findings Using BREAST-Q

## DISCUSSION

Since BREAST-Q was developed in 2009, it has been used extensively to assess the impact of oncoplastic treatment on PROs in breast surgery. PROMs questionnaires need to demonstrate reliability (ability to produce consistent and reproducible scores) and validity (ability to measure what is intended to be measured). Satisfying these requirements, the BREAST-Q has become the gold standard PROMs instrument for breast surgery.

The value of BREAST-Q is that clinicians now have data, with regard to patient satisfaction and HRQoL, which can be drawn upon to support patients in making decisions about best options for oncoplastic breast surgery. However, although results presented here give an indication of what might offer the best outcomes from a patient perspective, conclusions on PROs associated with breast oncological surgery should be interpreted with caution. Nearly all studies included in this review were observational studies with a small number of prospective and longitudinal in design, which meant the majority were classified as level III evidence. Different oncoplastic procedures alongside differences in study design and outcome reporting prevented a meta-analysis. This is not a criticism of the BREAST-Q, which has provided a fully validated patient-centered objective outcome measure, but a reflection on the fact that only 2.2% of plastic surgery articles are level I evidence.^15^ Given the nature of outcomes research in this population, it is recognized that the use of an interventional study design would be challenging and perhaps unethical or unfeasible. Lower levels of evidence can address some subtleties of surgical technique valuable to the practicing surgeon but not currently amenable to prospective, controlled, randomized trials.^16^

The BREAST-Q has been used as a national outcome measure in the National Mastectomy and Breast Reconstruction Audits in the United Kingdom (over 8,000 women between 3 and 18 months after surgery, with an 85% response rate),^17^ and the Army Of Women Study (over 7,600 women with a mean of 6.7 years since surgery and an 82% response rate) in the United States.^18^ It has also been used in the assessment of the surgical treatment of early stage breast cancer at a national level in the National Cancer database of the American College of Surgeons.^19^ Capturing PROMs data are recommended as a standard of care by our representative national bodies the Association of Breast Surgeons, the British Association of Plastic Reconstructive & Aesthetic Surgeons and the Royal College of Surgeons in the United Kingdom, and the American Society of Plastic Surgeons internationally. In addition, the BREAST-Q Satisfaction with Breasts scales is recommended in the International Consortium for Health Outcomes Measurement (ICHOM) standard order set for breast cancer patients.^20^

Traditionally, outcomes in oncoplastic breast surgery have been centered on the provider’s perspective, focusing on measuring complications and considering photographic analyses/panel assessments. Today, however, this is no longer sufficient to support progress being made in the field. In the current environment of the health care industry restrictions and performance metrics, quality-of-life outcomes are ever more important in clinical practice, research, and health care funding.

Perhaps the most useful setting of the BREAST-Q is in longitudinal studies. A single measurement provides little information as the results of surgical intervention change over time. Satisfaction with appearance between expander/implant reconstruction may be similar up to 3 years postoperatively, but the disparity with autologous reconstruction increases continuously with time, with free flap-based reconstruction being superior with regard to long-term outcome.^21^

Data on outcome measures within these studies should be used as a baseline, with outcomes from further prospective studies using BREAST-Q being used to strengthen evidence on best approaches to oncoplastic breast surgery. Future research is recommended and needed in the form of well-designed prospective longitudinal multicenter cohort or clinical interventional studies in large populations to determine the effectiveness of specific breast surgery types. Findings would further improve clinician decision making on which type of breast surgery should be advocated and adopted to enhance HRQoL and patients’ satisfaction.

It is worth noting that among the 54 articles included in this review, 10 were subanalyses of the Mastectomy Reconstruction Outcomes Consortium (MROC) Study. The MROC is a multicenter study involving 57 plastic surgeons at 11 academic and private practice sites across the United States and Canada. The primary aim of the MROC Study was to compare patient outcomes among the common surgical options for breast reconstruction. BREAST-Q was given to patients before reconstruction surgery. So far, 2-year outcomes have been partially reported (**Supplemental Digital Content 2** articles 28 and 52) A full analysis of MROC with longer term follow-up data will provide an important insight for the effectiveness of different surgical procedures on PROMs.

The key to the success in the surgical care of breast cancer patients is to make PROMs assessment a standard of care, to be completed before and after surgery at intervals, and to ensure a high questionnaire completion rate, essential to any high-quality study. An unpublished interim analysis of iBRA national practice suggests that only 7–13% of U.K. units are collecting PROMs prospectively. This is largely due to the current system being paper-based, manpower-dependent, and too labor-intensive to integrate into daily practice. As the world becomes increasingly digitized, use of electronic, emailed versions of the questionnaire, or completed via an App are likely to improve completion rates. It is on this basis, and in combination with well-designed high-level studies, that we will be able to use such data to make patient-centered, objective assessments of what is in the patient’s best interests, which will support fully informed shared decision making. Such implementation will give real-time updates on how patients feel, provide quality assurance, facilitate clinical decision making and evaluation of new treatments, provide surgeon feedback, audit data, and support health care funding based on quality of life.

### Study Limitations

Although systematic reviews have their own merit for increasing the statistical power of the existing small sample size of individual studies, they often present limitations. These include publication bias, language restrictions, heterogeneity across studies, and coding of key words. However, we adopted a well-structured search strategy, supplemented all “explode” functions and utilized hand searches and contacted breast surgical specialists to minimize the potential bias.

Another limitation is that the substantial heterogeneity in oncoplastic procedures alongside a variety of selected PROMs domains prevented us from performing a pooled analysis. Nevertheless, the aim and objectives of this current systematic review was to identify the updated evidence, and to make recommendations for future research implementing BREAST-Q for better management of the breast cancer population.

## CONCLUSIONS

Current evidence showed that BREAST-Q can effectively measure patient’s satisfaction and HRQoL in relation to different type of breast oncoplastic surgeries. BREAST-Q may be used as a tool to help clinicians and patients to make a decision on which breast surgery should be advocated and adopted to enhance HRQoL and patients’ satisfaction. Future more well-designed prospective multicenter cohort studies with longitudinal design using large sample populations or clinical interventional studies if feasible will provide further insight on the clinical application of BREAST-Q.

## ACKNOWLEDGMENTS

Authors thank Dr. Andrea Pusic who kindly provided relevant articles for this review and helped revise the draft.

## Supplementary Material
