# Supplementary material for: BREAST-Q Measurement of the Patient Perspective in Oncoplastic Breast Surgery: A Systematic Review
Source: Plast Reconstr Surg Glob Open. 2018 Aug 7;6(8):e1904. doi: 10.1097/GOX.0000000000001904 (PMC6143323; doi:10.1097/GOX.0000000000001904)
Supplement: Supplementary file 1 [file gox-6-e1904-s001.pdf]

SDC 1 Grading criteria for primary studies

Modified from Oxford evidence based Medicine (CEMB) guideline and Clinical Guidance Outcomes Group 2011 “Levels of Evidence and Clinical Guidance Outcomes group”

Grade I (strong evidence) --- G*ood quality of RCTs*

IA RCT with appropriate randomisation method, blinding, following up >80% and calculation of sample size

IB One or more above criteria

IC None of above criteria

Grade II (fairly strong evidence) ---*Non-RCT (CCT) or prospective cohort studies*

IIA Non-RCT or prospective cohort studies with follow up>75%, key characteristics matched between comparison groups, and the effects of important confounding variables adjusted

IIB One or more above criteria

IIC None of above criteria

Grade III (weaker evidence)— *Pre-post studies, retrospective cohort studies, cross-sectional cohort studies*

Grade IV (weak evidence) ----*Case-series, case-control studies*
