# Supplementary material for: BREAST-Q Measurement of the Patient Perspective in Oncoplastic Breast Surgery: A Systematic Review
Source: Plast Reconstr Surg Glob Open. 2018 Aug 7;6(8):e1904. doi: 10.1097/GOX.0000000000001904 (PMC6143323; doi:10.1097/GOX.0000000000001904)
Supplement: Supplementary file 2 [file gox-6-e1904-s002.pdf]

SDC2 Characteristics of all 54 articles included in this review

Author	Country	Sample Details	Comparison	Role of investigator	Type of study	Quality Assessment
1. Albornoz et al 2014	USA & Canada	633 women who had implant-based breast reconstruction with mean age of 51.3 yrs	Radiation versus no radiation	Observational	cross-sectional survey	Level III
2. Atisha et al 2015	USA	7,619 women who had a history of breast cancer surgery with mean age of 57.9 years ±9.4	Breast-conservation surgery with radiation versus mastectomy with or without reconstruction.	Observational	cross-sectional survey	Level III
3. Buchanan et al 2016	USA	30 patients had mastectomy with reconstruction and aged 49.1yrs ranging from 34yrs to 69yrs old	Unilateral mastectomy vs contralateral prophylactic mastectomy	Observational	Retrospective cohort	Level III
4. Bykowski et al 2017	USA	107 patients who underwent breast cancer-related Nipple-areola complex reconstruction	Before versus after Nipple-areola complex reconstruction; Follow up<1.5yr versus >2.5 yrs	Observational	cross-sectional survey	Level III

SDC2-2 Characteristics of all 54 articles included in this review

Author	Country	Sample Details	Comparison	Role of investigator	Type of study	Quality Assessment
[5.] Chao et al 2014	Taiwan	110 women aged 48.8 ± 9.5 yrs (range 27-71 yrs) who underwent mastectomy and/or breast reconstruction	Mastectomy with reconstruction vs without; immediate versus delayed reconstruction	Observational	Prospective cohort	Level IA
[6.] Davis et al 2014	USA	65 women who had breast reconstruction aged 49 years old	Multiple comparison	Observational	cross-sectional survey	Level III
[7.] de Blacam et al 2016	Ireland	61 patients aged 50yr±10 who had mastectomy with reconstruction	multiple regression	Observational	cross-sectional survey	Level III
[8.] Dieterich et al 2015	Germany	48 women aged 49.3±8.1vwho had IBBR with TiLOOP bra, 42 women aged 52.9± 8.6 had IBBR alone	IBBR alone versus IBBR with TiLOOP® Bra	Observational	cross-sectional survey	Level III
[9.] Duraes et al 2016	USA	176 patients who were diagnosed breast cancer	Immediate vs delayed vs secondary reconstruction	Observational	cross-sectional survey	Level III

IBBR=Implant-Base Breast Reconstruction

SDC2-3 Characteristics of all 54 articles included in this review

Author	Country	Sample Details	Comparison	Role of investigator	Type of study	Quality Assessment
10. Eltahir et al 2013	Netherland	137 women aged 50.5 years old who had mastectomy with/without reconstruction	Mastectomy alone versus mastectomy plus reconstruction	Observational	cross-sectional survey	Level III
11. Goyal et al 2011	UK	21 women aged 48yrs old (range 30–70).who had dermal sling-assisted breast reconstruction.	Multiple regression	Observational	cross-sectional survey	Level III
12. Ho et al 2013	USA	510 women aged 54.3 ± 9.3 yrs old who had breast reconstruction	Multiple regression	Observational	cross-sectional survey	Level IIB
13. Howes et al 2016	Australia	400 women age 54.3 yrs who had breast cancer surgery	BCS vs. mastectomy with or without reconstruction	Observational	cross-sectional survey	Level III
14. Hwang et al 2016	USA	3977 women had breast cancer surgery with mean age of 56.5yrs	CPM versus. no CPM	Observational	cross-sectional survey	Level III

BCS=Breast Conserving Surgery ; CPM= contralateral prophylactic mastectomies

SDC2-4 Characteristics of all 54 articles included in this review

Author	Country	Sample Details	Comparison	Role of investigator	Type of study	Quality Assessment
[15]. Inbal et al 2012	Israel	51 patients had unilateral breast reconstruction using the DIEP flap with mean age of 49.7yrs	Late vs. concomitantly contralateral breast adjustment vs No contralateral adjustment	Observational	cross-sectional survey	Level III
[16]. Jeevan et al 2014	UK	7110 women had mastectomy with or without reconstruction	Mastectomy alone vs mastectomy with breast reconstruction	Observational	Prospective cohort	Level IIA
[17]. Khavanni et al 2018	USA &Canada	822 patients aged 48.0 ± 10.3 yrs old who undergoing immediate or delayed breast reconstruction	Shaped and round silicone gel implant	Observational	Prospective cohort	Level IIA
[18]. Koslow et al 2013	USA	294 patients had mastectomy and implant-based reconstruction with mean age of 48.4 yrs	CPM vs. Non-CPM	Observational	Retrospective Case control	Level IV

CPM= contralateral prophylactic mastectomies; DIEP= deep inferior epigastric perforator

SDC2-5 Characteristics of all 54 articles included in this review

Author	Country	Sample Details	Comparison	Role of investigator	Type of study	Quality Assessment
[19.] Kulkarni et al 2018	USA &Canada	2667 women aged 49,7 yrs old who had breast reconstruction	5 type of reconstruction procedures	Observational	Prospective cohort	Level IIA
[20.] Liu et al 2014	USA	74 women aged 49.1yrs underwent immediate unilateral breast reconstruction	Expander implant vs microsurgical abdominal flap breast reconstruction	Observational	Retrospective case-controlled	Level IV
[21.] Ludolph et al 2015	Germany	179 patients aged 56yrs who had free autologous breast reconstruction	Reconstruction with DIEP versus ms-TRAM flap	Observational	cross-sectional survey	Level III
[22.] Macadam[a] et al 2013	Canada	143 women aged 53.9 yrs who had implant-based breast reconstruction	Silicone implant versus saline implant	Observational	cross-sectional survey	Level III
[23.] Macadam[b] et al 2013	Canada	128 patients aged 51.9 yrs who had alloplastic breast reconstruction	Shaped vs round silicone gel implant	Observational	cross-sectional survey	Level III
[24.] McCarthy et al 2010	USA &Canada	482 patients aged 52.5 yrs who had implant-breast reconstruction	Silicone implant versus saline implant	Observational	cross-sectional survey	Level III

DIEP= deep inferior epigastric perforator; ms-TRAM=Muscle-sparing transverse rectus abdominis myocutaneous

SDC2-6 Characteristics of all 54 articles included in this review

Author	Country	Sample Details	Comparison	Role of investigator	Type of study	Quality Assessment
25. McCarthy et al 2014	USA	308 women had mastectomy with or without breast reconstruction (61 yrs old mastectomy alone, 52 yrs old autogenous reconstruction, 50 yrs old Implant reconstruction)	Mastectomy alone versus immediate autogenous reconstruction or two-stage tissue expander /implant reconstruction	Observational	cross-sectional survey	Level III
26. Ng SK et al 2016	Australia	143 women aged 54.5 ± 12.9 years who had therapeutic or prophylactic mastectomy	Mastectomy alone versus immediate or delayed reconstruction	Observational	cross-sectional survey	Level III
27. Rosson et al 2013	USA	170 Woman aged 50.3 ±9.5 undergoing immediate or delayed reconstruction or those who had reconstruction before but need major revisions	Immediate reconstruction vs delayed vs major revision reconstruction	Observational	cross-sectional survey	Level III

SDC2-7 Characteristics of all 54 articles included in this review

Author	Country	Sample Details	Comparison	Role of investigator	Type of study	Quality Assessment
28. Simpson et al 2014	Canada	13 patients aged 50.8 yrs old undergoing breast reconstruction	Immediate reconstruction vs delayed reconstruction	Observational	Retrospective Case control	Level IV
29. Sinha et al 2016	Australia	101 patients mean aged 47yrs who had autologous microsurgical free-flap breast reconstruction with an abdominal donor site	Normal-weight versus overweight versus obese patients	Observational	cross-sectional survey	Level III
30. Sisco et al 2015	USA	214 women had mastectomy with or without reconstruction with mean age of 60.5 yrs old.	Mastectomy alone versus mastectomy with breast reconstruction	Observational	cross-sectional survey	Level III
31. Song et al 2016	USA & Canada	950 patients underwent autologous reconstruction with average age of 58.2 yrs	Older than 65 yrs versus younger than 65yrs	Observational	cross-sectional survey	Level III

SDC2-8 Characteristics of all 54 articles included in this review

Author	Country	Sample Details	Comparison	Role of investigator	Type of study	Quality Assessment
[32.] Sugrue et al 2013	Ireland	30 women aged 43 ± 11 yrs who had mastectomy and immediate reconstruction	Pre-operation vs post--operation	Observational	cross-sectional survey	Level III
[33.] Susarla et al 2015	USA	268 patients had single-staged or two-staged implant (aged 47.2± 10.3 yrs and 47.6± 10.2 yrs respectively)	Single-staged implant vs two-staged implant based reconstruction	Observational	cross-sectional survey	Level III
[34.] Wechman et al 2015	USA &Canada	2,013 women aged 51.1yrs undergoing primary breast reconstruction	7 types of reconstruction procedures	Observational	prospective cohort	Level IIB
[35.] Wei et al 2016	USA	254 patients had SSM aged 44.9± 9.4 yrs and NSM aged 45.7 ± 7.9 yrs	NSM vs SSM	Observational	Retrospective cohort	Level IV
[36.] Wu et al 2013	USA	76 women underwent breast reconstruction	HADM versus without HADM	Interventional	prospective non-RCT	Level IIA

NSM=Nipple-sparing mastectomy; SSM=skin-sparing mastectomy

SDC2-9 Characteristics of all 54 articles included in this review

Author	Country	Sample Details	Comparison	Role of investigator	Type of study	Quality Assessment
[37.] Zhong et al 2016	Canada	106 women undergoing microsurgical autologous reconstruction	Immediate autologous tissue reconstruction vs delayed reconstruction	Observational	Prospective cohort	Level IIA
[38.] Bailey et al 2017	USA	64 woman underwent mastectomy	Nipple-sparing or non-nipple sparing mastectomy	Observational	Retrospective Case control	Level IV
[39.] Bennett et al 2017	USA &Canada	2048 woman had breast mound reconstruction	Fat grafting versus no fat grafting reconstruction	Observational	prospective cohort	Level IIA
[40.] Broecker et al 2017	USA	87 high stage breast cancer patients underwent BCS	BCS alone vs BCS with immediate oncoplastic reduction	Observational	Retrospective Case control	Level IV
[41.] Brown et al 2017	Australia	46 woman had AFG after reconstruction or BCS	Reconstruction versus BCS	Observational	cross-sectional survey	Level III
[42.] Chand et al 2017	UK	155 breast cancer patients	Therapeutic mammoplasty versus LDI	Observational	cross-sectional survey	Level III

AFG, Autologous fat grafting; BCS=Breast conservation surgery; DTI, Direct to implant; LDI LDI= latissimus dorsi implant

SDC2-10 Characteristics of all 54 articles included in this review

Author	Country	Sample Details	Comparison	Role of investigator	Type of study	Quality Assessment
[43.] Cogliandro[a] et al 2017	Italy	70 patients underwent breast reconstruction with or without delayed lipofilling	delayed lipolifiting versus without lipolifting	Observational	Retrospective cohort	Level III
[44.] Cogliandro[b] et al 2017	Italy	55 patients had monolateral mastectomy with breast implant and contralateral adjustment	Crescent mastopexy versus other mastopexy technique	Observational	cross-sectional survey	Level III
[45.] Cornelissen et al 2017	Netherland	32 patients underwent DIEP breast reconstruction	With nerve coaptation versus without	Observational	Retrospective cohort study	Level III
[46.] Erdmann-Sager et al 2017.	USA & Canada	720 patients had abdominal-based-reconstruction	4 types of autologous reconstruction procedures	Observational	Prospective cohort	Level IIA
[47].Ménez et al 2017	France	123 women had autologous breast reconstruction	DIEP versus LDI versus ALD	Observational	cross-sectional survey	Level III
[48.] Pusic et al 2017	USA &Canada	1632 patients had immediate reconstruction	implant versus autologous reconstruction	Observational	Prospective cohort	Level IIA

ALD=Autologous latissimus dorsi; DIEP= deep inferior epigastric perforator; LDI= latissimus dorsi implant

SDC2-11 Characteristics of all 54 articles included in this review

Author	Country	Sample Details	Comparison	Role of investigator	Type of study	Quality Assessment
49. Pont et al 2017	Italy	230 patients underwent mastectomy with immediate free flap reconstruction	Adjuvant radiotherapy versus without radiotherapy	Observational	Retrospective cohort	Level III
50. Qureshi et al 2017	USA	59 patients undergoing NSM procedures with immediate breast reconstruction	Direct-to-Implant versus Tissue Expander/Implant	Observational	Retrospective cohort	Level III
51. Sorkin et al 2018	USA & Canada	1297 women underwent immediate reconstruction with tissue expander	With acellular Dermal Matrix versus without	Observational	Prospective cohort	Level IIA
52. Srinivasa et al 2017	USA & Canada	1427 women had immediate reconstruction	Direct-to-Implant versus Tissue Expander/Implant	Observational	Prospective cohort	Level IIA
53.Thorarinsson et al .2017	Sweden	459 patients had delayed breast reconstruction	4 types of reconstruction procedures	Observational	Retrospective cohort	Level IV
54. Yoon et al 2018	USA &Canada	1957 patients breast reconstruction	Immediate versus delayed reconstruction	Observational	Prospective cohort	Level IIA

1. Albornoz CR, Matros E, McCarthy CM et al. Implant breast reconstruction and radiation: a multicentre analysis of long-term health-related quality of life and satisfaction. *Ann Surg Oncol* (2014) 21: 2159.
2. Atisha DM, Rushing CN, Samsa GP et al. A national snapshot of satisfaction with breast cancer procedures. *Ann Surg Oncol.* 2015;22:361-9.
3. Buchanan PJ, Abdulghani M, Waljee JF et al. An Analysis of the Decisions Made for Contralateral Prophylactic Mastectomy and Breast Reconstruction. *Plast Reconstr Surg.* 2016;138:29-40.
4. Bykowski MR, Emelife PI, Emelife NN et al. Nipple-areola complex reconstruction improves psychosocial and sexual well-being in women treated for breast cancer. *J Plast Reconstr Aesthetic Surg.* 2017;70:209-214.
5. Chao LF, Patel KM, Chen SC et al. Monitoring patient-centered outcomes through the progression of breast reconstruction: a multicentered prospective longitudinal evaluation. *Breast Cancer Res Treat.* 2014;146:299-308.
6. Davis GB, Lang JE, Peric M et al. .Breast Reconstruction Satisfaction Rates at a Large County Hospital. *Ann Plast Surg*. 2014;72 Suppl 1:S61-5.
7. de Blacam C., Healy C., Quinn L et al. Is satisfaction with surgeon a determining factor in patient reported outcomes in breast reconstruction? *Journal of Plastic, Reconstructive and Aesthetic Surgery,* 2016:69:9:1248-53.
8. Dieterich M, Angres J, Stachs A et al. Patient-Report Satisfaction and Health-Related Quality of Life in TiLOOP® Bra-Assisted or Implant-Based Breast Reconstruction Alone. Aesthetic Plastic Surgery 2015;39(4):523-533.

9. Duraes EFR, Durand P, Duraes LC et al. Comparison of preoperative quality of life in breast reconstruction, breast aesthetic and non-breast plastic surgery patients: A cross-sectional study. *Journal of Plastic, Reconstructive & Aesthetic Surgery: JPRAS* 2016;69(11):1478-85.
10. Eltahir Y, Werners LL, Dreise MM et al. Quality-of-life outcomes between mastectomy alone and breast reconstruction: comparison of patient-reported BREAST-Q and other health-related quality-of-life measures. *Plast Reconstr Surg.* 2013;132(2):201e-209e.
11. Goyal A, Wu JM, Chandran VP et al. Outcome after autologous dermal sling-assisted immediate breast reconstruction. *The British Journal of Surgery* 2011;98(9):1267-72.
12. Ho AL, Klassen AF, Cano S, Scott AM, Pusic AL. Optimizing patient-centered care in breast reconstruction: the importance of preoperative information and patient-physician communication. *Plast Reconstr Surg.* 2013;132(2):212e-220e.
13. Howes BHL, Watson DI, Xu C et al. Quality of life following total mastectomy with and without reconstruction versus breast-conserving surgery for breast cancer: A case-controlled cohort study. *Journal of Plastic, Reconstructive & Aesthetic Surgery: JPRAS* 2016;69(9):1184-91.
14. Hwang ES, Locklear TD, Rushing CN et al. Patient-Reported Outcomes After Choice for Contralateral Prophylactic Mastectomy. *Journal of Clinical Oncology* 2016;34(13):1518-27.
15. Inbal A, Gur E, Otremski E et al. Simultaneous contralateral breast adjustment in unilateral deep inferior epigastric perforator breast reconstruction. *J Reconstr Microsurg.* 2012;28(5):285-92.

16. Jeevan, R., Cromwell, D.A., Browne, J.P. et al, Findings of a national comparative audit of mastectomy and breast reconstruction surgery in England. *J Plast Reconstr Aesthet Surg.* 2014;67:1333–44.
17. Khavanin, Nima.; Clemens, Mark W. et al. Shaped versus Round Implants in Breast Reconstruction: A Multi-Institutional Comparison of Surgical and Patient-Reported Outcomes. *Plastic and Reconstructive Surgery:* 2017;139:1063–70.
18. Koslow S1, Pharmer LA, Scott AM et al. Long-term patient-reported satisfaction after contralateral prophylactic mastectomy and implant reconstruction. *Ann Surg Oncol.* 2013;20(11):3422-9
19. Kulkarni AR, Pusic AL, Hamill JB et al. Factors associated with acute postoperative pain following breast reconstruction. *JPRAS Open* 2017;11: 1-13.
20. Liu C, Zhuang Y, Momeni A et al. Quality of life and patient satisfaction after microsurgical abdominal flap versus staged expander/implant breast reconstruction: a critical study of unilateral immediate breast reconstruction using patient-reported outcomes instrument BREAST-Q. *Breast Cancer Res Treat.* 2014;146(1):117-26.
21. Ludolph I, Horch RE, Harlander M et al. Is there a Rationale for Autologous Breast Reconstruction in Older Patients? A Retrospective Single Center Analysis of Quality of life, Complications and Comorbidities after DIEP or ms-TRAM Flap Using the BREAST-Q. *Breast J.* 2015;21(6):588-95
22. Macadam[a] SA, Ho AL, Cook EF, Jr., Lennox PA, Pusic AL. Patient satisfaction and health-related quality of life following breast reconstruction: patient-reported outcomes among saline and silicone implant recipients. *Plast Reconstr Surg.* 2010;125(3):761-71.

23. Macadam SA, Ho AL, Lennox PA, Pusic AL. Patient-reported satisfaction and health-related quality of life following breast reconstruction: a comparison of shaped cohesive gel and round cohesive gel implant recipients. *Plast Reconstr Surg.* 2013;131(3):431-41.
24. McCarthy CM, Klassen AF, Cano SJ et al. Patient satisfaction with postmastectomy breast reconstruction: a comparison of saline and silicone implants. *Cancer*. 2010;116(24):5584-91.
25. McCarthy CM, Mehrara BJ, Long T et al. Chest and upper body morbidity following immediate postmastectomy breast reconstruction. *Ann Surg Oncol.* 2014;21(1):107-12..
26. Ng SK, Hare RM, Kuang RJ et al. Breast Reconstruction Post Mastectomy: Patient Satisfaction and Decision Making. *Ann Plast Surg.* 2016;76(6):640-4.
27. Rosson GD, Shridharani SM, Magarakis M et al. Quality of life before reconstructive breast surgery: A preoperative comparison of patients with immediate, delayed, and major revision reconstruction. *Microsurgery.* 2013;33(4):253-8.
28. Simpson JS., Baltzer H., McMillian CR et al. Multidisciplinary assessment for immediate breast reconstruction: A new approach. *Surgical Practice,* 2014;18: 111–16.
29. Sinha S, Ruskin O, D'Angelo A et al. Are overweight and obese patients who receive autologous free-flap breast reconstruction satisfied with their postoperative outcome? A single-centre study. *J Plast Reconstr Aesthet Surg.* 2016;69(1):30-6.
30. Sisco M, Johnson DB, Wang C et al.. The quality-of-life benefits of breast reconstruction do not diminish with age. *J Surg Oncol.* 2015;111(6):663-8.

31. Song D, Slater K, Papsdorf M et al. Autologous Breast Reconstruction in Women Older Than 65 Years Versus Women Younger Than 65 Years: A Multi-Center Analysis. *Ann Plast Surg.* 2016;76(2):155-63.
32. Sugrue R, MacGregor G, Sugrue M et al. An evaluation of patient reported outcomes following breast reconstruction utilizing BREAST-Q. *Breast.* 2013;22(2):158-61.
33. Susarla SM1, Ganske I, Helliwell L et al. Comparison of clinical outcomes and patient satisfaction in immediate single-stage versus two-stage implant-based breast reconstruction. *Plast Reconstr Surg.* 2015;135(1):1e-8e.
34. .Wei CH, Scott AM, Price AN et al. Psychosocial and Sexual Well-Being Following Nipple-Sparing Mastectomy and Reconstruction. *The Breast Journal* 2016;22(1):10-17.
35. Weichman KE, Hamill JB, Kim HM, et al. Understanding the recovery phase of breast reconstructions: Patient-reported outcomes correlated to the type and timing of reconstruction. *J Plast Reconstr Aesthet Surg.* 2015;68(10):1370-8.
36. Wu C., Cipriano J., Osgood J. et al. "Human acellular dermal matrix (AlloDerm®) dimensional changes and stretching in tissue expander/implant breast reconstruction", *Journal of plastic, reconstructive & aesthetic surgery: JPRAS,* 2013; 66(10):1376-81.
37. Zhong T, Hu J, Bagher S et al. A Comparison of Psychological Response, Body Image, Sexuality, and Quality of Life between Immediate and Delayed Autologous Tissue Breast Reconstruction: A Prospective Long-Term Outcome Study. *Plastic and Reconstructive Surgery* 2016;138(4):772-80.
38. Bailey CR, Ogbuagu O, Baltodano PA et al. Quality-of-Life Outcomes Improve with Nipple-Sparing Mastectomy and Breast Reconstruction. *Plast Reconstr Surg.* 2017;140(2):219-26.

39. Bennett KG, Qi J, Kim HM, Hamill JB, Wilkins EG, Mehrara BJ, Kozlow JH. Association of Fat Grafting With Patient-Reported Outcomes in Postmastectomy Breast Reconstruction. *JAMA Surg.* 2017;152(10):944-50.

40. Broecker JS, Hart AM, Styblo TM et al. Neoadjuvant Therapy Combined With Oncoplastic Reduction for High-Stage Breast Cancer Patients. *Ann Plast Surg.* 2017;78(6S Suppl 5):S258-S262.

41. Brown AWW, Kabir M, Sherman KA et al. Patient reported outcomes of autologous fat grafting after breast cancer surgery. *Breast.* 2017;35:14-20.

42. Chand ND, Browne V, Paramanathan N et al. Patient-Reported Outcomes Are Better after Oncoplastic Breast Conservation than after Mastectomy and Autologous Reconstruction. *Plast Reconstr Surg Glob Open.*2017;24;5(7):e1419.

43. Cogliandro A, Brunetti B, Barone M et al. Management of contralateral breast following mastectomy and breast reconstruction using a mirror adjustment with crescent mastopexy technique. *Breast Cancer.* 2018;25(1):94-99.

44. Cogliandro A, Barone M, Tenna S, Morelli Coppola M, Persichetti P. The Role of Lipofilling After Breast Reconstruction: Evaluation of Outcomes and Patient Satisfaction with BREAST-Q. *Aesthetic Plast Surg.* 2017;41(6):1325-31.

45. Cornelissen AJM, Beugels J, van Kuijk SMJ et al. Sensation of the autologous reconstructed breast improves quality of life: a pilot study. *Breast Cancer Res Treat.* 2017. Ahead of print

46. . Erdmann-Sager J, Wilkins EG, Pusic AL et al. Complications and Patient-Reported Outcomes after Abdominal-Based Breast Reconstruction: Results of the Mastectomy Reconstruction Outcomes Consortium (MROC) Study. *Plast Reconstr Surg.* 2017, Ahead of print

47. Ménez T, Michot A, Tamburino S et al. Multicenter evaluation of quality of life and patient satisfaction after breast reconstruction, a long-term retrospective study. *Ann Chir Plast Esthet.* 2017. Ahead of print

48. Pont LP, Marcelli S, Robustillo M et al. Immediate Breast Reconstruction with Abdominal Free Flap and Adjuvant Radiotherapy: Evaluation of Quality of Life and Outcomes. *Plast Reconstr Surg.* 2017;140(4):681-90.

49. Pusic AL, Matros E, Fine N et al. Patient-reported outcomes 1 year after immediate breast reconstruction: Results of the Mastectomy Reconstruction Outcomes Consortium study. *J Clin Oncol* . 2017, 1;35(22):2499-06.

50. Qureshi AA, Odom EB, Parikh RP et al. Patient-Reported Outcomes of Aesthetics and Satisfaction in Immediate Breast Reconstruction After Nipple-Sparing Mastectomy With Implants and Fat Grafting. *Aesthet Surg J.* 2017; 1;37(9):999-1008.

51. Sorkin M, Qi J, Kim HM et al. Acellular Dermal Matrix in Immediate Expander/Implant Breast Reconstruction: A Multicenter Assessment of Risks and Benefits. *Plast Reconstr Surg.* 2017;140(6):1091-1100.

52. Srinivasa DR, Garvey PB, Qi J, Hamill JB, Kim HM, Pusic AL, Kronowitz SJ, Wilkins EG, Butler CE, Clemens MW. Direct-to-Implant versus Two-Stage Tissue Expander/Implant Reconstruction: 2-Year Risks and Patient-Reported Outcomes from a Prospective, Multicenter Study. *Plast Reconstr Surg.* 2017;140(5):869-877.

53. Thorarinsson A, Fröjd V, Kölby L et al. Long-Term Health-Related Quality of Life after Breast Reconstruction: Comparing 4 Different Methods of Reconstruction. *Plast Reconstr Surg Glob Open.* 2017;5(6):e1316.

54. Yoon AP, Qi J, Brown DL et al. Outcomes of immediate versus delayed breast reconstruction: Results of a multicenter prospective study. *Breast.* 2018;37:72-79.
